# Supplementary material for: Tracing the development and lifespan change of population-level structural asymmetry in the cerebral cortex
Source: eLife. 2023 Jun 19;12:e84685. doi: 10.7554/eLife.84685 (PMC10368427; doi:10.7554/eLife.84685)
Supplement: MDAR checklist [file elife-84685-mdarchecklist1.docx]

**Materials Design Analysis Reporting (MDAR)**

**Checklist for Authors**

The [MDAR framework](https://osf.io/xfpn4/) establishes a minimum set of requirements in transparent reporting mainly applicable to studies in the life sciences.

*eLife* asks authors to **provide detailed information within their article** to facilitate the interpretation and replication of their work. Authors can also upload supporting materials to comply with relevant reporting guidelines for health-related research (see [EQUATOR Network](http://www.equator-network.org/%20)), life science research (see the [BioSharing Information Resource](http://biosharing.org/)), or animal research (see the [ARRIVE Guidelines](http://www.plosbiology.org/article/info:doi/10.1371/journal.pbio.1000412) and the [STRANGE Framework](https://doi.org/10.1038/d41586-020-01751-5); for details, see *eLife*’s [Journal Policies](https://reviewer.elifesciences.org/author-guide/journal-policies)). Where applicable, authors should refer to any relevant reporting standards materials in this form.

For all that apply, please note **where in the article** the information is provided. Please note that we also collect information about data availability and ethics in the submission form.

**Materials:**

| **Newly created materials** | **Indicate where provided: section/figure legend** | **N/A** |
| --- | --- | --- |
| The manuscript includes a dedicated "materials availability statement" providing transparent disclosure about availability of newly created materials including details on how materials can be accessed and describing any restrictions on access. |  | X |
|  |  |  |
| **Antibodies** | **Indicate where provided: section/figure legend** | **N/A** |
| For commercial reagents, provide supplier name, catalogue number and [RRID](https://scicrunch.org/resources), if available. |  | X |
|  |  |  |
| **DNA and RNA sequences** | **Indicate where provided: section/figure legend** | **N/A** |
| Short novel DNA or RNA including primers, probes: Sequences should be included or deposited in a public repository. |  | X |
|  |  |  |
| **Cell materials** | **Indicate where provided: section/figure legend** | **N/A** |
| Cell lines: Provide species information, strain. Provide accession number in repository OR supplier name, catalog number, clone number, OR RRID. |  | X |
| Primary cultures: Provide species, strain, sex of origin, genetic modification status. |  | X |
|  |  |  |
| **Experimental animals** | **Indicate where provided: section/figure legend** | **N/A** |
| Laboratory animals or Model organisms: Provide species, strain, sex, age, genetic modification status. Provide accession number in repository OR supplier name, catalog number, clone number, OR RRID. |  | X |
| Animal observed in or captured from the field: Provide species, sex, and age where possible. |  | X |
|  |  |  |
| **Plants and microbes** | **Indicate where provided: section/figure legend** | **N/A** |
| Plants: provide species and strain, ecotype and cultivar where relevant, unique accession number if available, and source (including location for collected wild specimens). |  | X |
| Microbes: provide species and strain, unique accession number if available, and source. |  | X |
|  |  |  |
| **Human research participants** | **Indicate where provided: section/figure legend) or state if these demographics were not collected** | **N/A** |
| If collected and within the bounds of privacy constraints report on age, sex, gender and ethnicity for all study participants. | Sample demographics for each analysis (including age and sex) are provided in Supplementary file 1A. Gender and ethnicity are not provided as this information was not available for many cohorts. |  |

**Design:**

| **Study protocol** | **Indicate where provided: section/figure legend** | **N/A** |
| --- | --- | --- |
| If the study protocol has been pre-registered, provide DOI. For clinical trials, provide the trial registration number OR cite DOI. |  | X |
|  |  |  |
| **Laboratory protocol** | **Indicate where provided: section/figure legend** | **N/A** |
| Provide DOI OR other citation details if detailed step-by-step protocols are available. |  | X |
|  |  |  |
| **Experimental study design (statistics details) *** | | |
| **For in vivo studies: State whether and how the following have been done** | **Indicate where provided: section/figure legend. If it could have been done, but was not, write “not done”** | **N/A** |
| Sample size determination | Not done |  |
| Randomisation |  | X |
| Blinding |  | X |
| Inclusion/exclusion criteria | Key references for inclusion/exclusion criteria for participation in each cohort are given in section 4.1.1. We applied no inclusion/exclusion criteria here |  |
|  |  |  |
| **Sample definition and in-laboratory replication** | **Indicate where provided: section/figure legend** | **N/A** |
| State number of times the experiment was replicated in the laboratory. |  | X |
| Define whether data describe technical or biological replicates. |  | X |
|  |  |  |
| **Ethics** | **Indicate where provided: section/submission form** | **N/A** |
| Studies involving human participants: State details of authority granting ethics approval (IRB or equivalent committee(s), provide reference number for approval. | Section 4.1 |  |
| Studies involving experimental animals: State details of authority granting ethics approval (IRB or equivalent committee(s), provide reference number for approval. |  | X |
| Studies involving specimen and field samples: State if relevant permits obtained, provide details of authority approving study; if none were required, explain why. |  | X |
|  |  |  |
| **Dual Use Research of Concern (DURC)** | **Indicate where provided: section/submission form** | **N/A** |
| If study is subject to dual use research of concern regulations, state the authority granting approval and reference number for the regulatory approval. |  | X |

**Analysis:**

| **Attrition** | **Indicate where provided: section/figure legend** | **N/A** |
| --- | --- | --- |
| Describe whether exclusion criteria were pre-established. Report if sample or data points were omitted from analysis. If yes, report if this was due to attrition or intentional exclusion and provide justification. | Section 4.1.3, Section 4.3.2, Figure 1 |  |
|  |  |  |
| **Statistics** | **Indicate where provided: section/figure legend** | **N/A** |
| Describe statistical tests used and justify choice of tests. | Section 4.3 |  |
|  |  |  |
| **Data availability** | **Indicate where provided: section/submission form** | **N/A** |
| For newly created and reused datasets, the manuscript includes a data availability statement that provides details for access (or notes restrictions on access). | Section 5 Data sharing/availability |  |
| When newly created datasets are publicly available, provide accession number in repository OR DOI and licensing details where available. |  | X |
| If reused data is publicly available provide accession number in repository OR DOI, OR URL, OR citation. | Section 5 Data sharing/availability |  |
|  |  |  |
| **Code availability** | **Indicate where provided: section/figure legend** | **N/A** |
| For any computer code/software/mathematical algorithms essential for replicating the main findings of the study, whether newly generated or re-used, the manuscript includes a data availability statement that provides details for access or notes restrictions. | Section 5 Data sharing/availability |  |
| Where newly generated code is publicly available, provide accession number in repository, OR DOI OR URL and licensing details where available. State any restrictions on code availability or accessibility. | Section 5 Data sharing/availability | X |
| If reused code is publicly available provide accession number in repository OR DOI OR URL, OR citation. |  | X |

**Reporting:**

The MDAR framework recommends adoption of discipline-specific guidelines, established and endorsed through community initiatives.

| **Adherence to community standards** | **Indicate where provided: section/figure legend** | **N/A** |
| --- | --- | --- |
| State if relevant guidelines (e.g., ICMJE, MIBBI, ARRIVE, STRANGE) have been followed, and whether a checklist (e.g., CONSORT, PRISMA, ARRIVE) is provided with the manuscript. |  | X |

* We provide the following guidance regarding transparent reporting and statistics; we also refer authors to [Ten common statistical mistakes to watch out for when writing or reviewing a manuscript](https://doi.org/10.7554/eLife.48175).

**Sample-size estimation**

- You should state whether an appropriate sample size was computed when the study was being designed
- You should state the statistical method of sample size computation and any required assumptions
- If no explicit power analysis was used, you should describe how you decided what sample (replicate) size (number) to use

For all analyses sample size was determined based on data availability. No formal analyses were performed to predetermine sample sizes. We gathered as much data as we could, from both cross-sectional and longitudinal (LCBC dataset only) observations, and included all observations meeting the stated criteria for each analysis (e.g. age-range). There was one exception to this: vertex-wise analysis for asymmetry effects in UK Biobank (UKB) were performed in a random sample of n=1000 UKB subjects. This is because the mean effects were detectable with high overlap in relatively small samples, and it was thus deemed unnecessary to perform this analysis in the full UKB sample (n ~40K). Therefore, for this analysis only, the UKB sample was restricted to be comparable in size to the other datasets (see section 4.1.1).

We aimed to replicate effects in 7 independent samples as starting point, then test replication of all downstream analyses either in the three largest samples, or in all datasets with available data to test the effect-of-interest (e.g. heritability effects were tested using both twin and genomic methods). Only the analysis of individual differences in UKB data did not permit a replication attempt.

**Replicates**

- You should report how often each experiment was performed
- You should include a definition of biological versus technical replication
- The data obtained should be provided and sufficient information should be provided to indicate the number of independent biological and/or technical replicates
- If you encountered any outliers, you should describe how these were handled
- Criteria for exclusion/inclusion of data should be clearly stated
- High-throughput sequence data should be uploaded before submission, with a private link for reviewers provided (these are available from both GEO and ArrayExpress)

Information regarding the number of independent samples used for each analysis can be found in section 4.1. We used already available datasets. We applied no inclusion/exclusion criteria prior to analyses, and used all available observations meeting the stated criteria per analysis. Cohort-specific references outlining general inclusion/exclusion criteria required for participation in each study are given in section 4.1. In general, the samples consisted of cognitively healthy individuals, with the exception of UKB, which is a population-based study of the UK. Information regarding outliers can be found in section 4.1.3 and 4.3.2 and Figure 5-figure supplement 4.

**Statistical reporting**

- Statistical analysis methods should be described and justified
- Raw data should be presented in figures whenever informative to do so (typically when N per group is less than 10)
- For each experiment, you should identify the statistical tests used, exact values of N, definitions of center, methods of multiple test correction, and dispersion and precision measures (e.g., mean, median, SD, SEM, confidence intervals; and, for the major substantive results, a measure of effect size (e.g., Pearson's r, Cohen's d)
- Report exact p-values wherever possible alongside the summary statistics and 95% confidence intervals. These should be reported for all key questions and not only when the p-value is less than 0.05.

All statistical analyses are reported in Section 4.3. Briefly, for analysis 1: asymmetry effects, we implemented vertex-wise linear mixed effect models (LME) in 7 independent samples. LME provided a common analysis framework to model both the mixed-effects LCBC sample with longitudinal data, and the repeated measures cross-sectional samples. For analysis 2: lifespan trajectories, we implemented Generalized Additive Mixed Models. This enabled flexibly fitting nonlinear models with relaxed assumptions of the shape of the lifespan trajectories, and allowed us to incorporate the full breadth of the cross-sectional and longitudinal data available through our lifespan sample, including repeat scans at a single timepoint. For analysis 3: interregional correlations, we employed Mantel tests to test matrix similarity between all dataset-pairs from the three largest adult samples. For analysis 4: heritability, we employed genome-based restricted maximum likelihood methods to assess SNP-based heritability and genetic correlations, and structural equation models (AE models) to assess twin-based heritability and genetic correlations. These differing heritability estimation methods enabled testing replication. For analysis 5: individual differences, we used the large population-based UK biobank study to test associations between asymmetry and several factors purportedly associated with it (cognition, handedness, sex, brain size). Only this analysis did not enable a replication attempt due to the unique size and scope of UKB data, and the small inter-indivdual effect sizes found.

Raw data is presented where informative and feasible (e.g. lifespan trajectories in Figs 2-3; individual-level asymmetry effects in Figure 1F,H)

Experimental details are available in section 4.1 and 4.3

Where possible, exact p-values are reported, except in four instances. Two of these refer to a non-significant effect tested in 3 replication samples that was unambiguously non-significant in all samples (both reported as “all p > .5”). The others refer to reports where the p-value attained maximal significance for the tested effect in the program used to test it (i.e. maximal significance for the Mantel tests (“ade4” R package) was p < 9.9e-5, maximal significance for R correlations = p < 2.2e-16). Confidence intervals are included in all figures and reporting where relevant.

**Group allocation**

- Indicate how samples were allocated into experimental groups (in the case of clinical studies, please specify allocation to treatment method); if randomization was used, please also state if restricted randomization was applied
- Indicate if masking was used during group allocation, data collection and/or data analysis

Not applicable as no group allocation was performed
